# High Capacity of Nutrient Accumulation by Invasive *Solidago canadensis* in a Coastal Grassland

**DOI:** 10.3389/fpls.2019.00575

**Published:** 2019-05-07

**Authors:** Xiao-Qi Ye, Ya-Nan Yan, Ming Wu, Fei-hai Yu

**Affiliations:** ^1^Research Station of Hangzhou Bay Wetland Ecosystems, National Forestry Bureau, Institute of Subtropical Forestry, Chinese Academy of Forestry, Hangzhou, China; ^2^College of Life Sciences, Shanxi Normal University, Linfen, China; ^3^College of Life Sciences, Taizhou University, Taizhou, China

**Keywords:** *Solidago canadensis*, coastal grassland, nutrient cycling, aboveground biomass, litter decomposition

## Abstract

**Background:**

*Solidago canadensis* is a notorious invasive species from North America that is spreading across East China. It is invading some coastal grasslands and replacing native grass species. The effects of the *S. canadensis* invasion on soil nutrient cycling in the grasslands remain unclear. This study examined the effects of the invasion of *S. canadensis* on macronutrient accumulation in species aboveground part and soil.

**Methods:**

Aboveground biomass, macronutrient (N, P, and K) pools in biomass, litter mass and decomposition rates, soil macronutrient availability and soil microbial biomass and enzyme activity that were related to nutrient transformation were compared between plots invaded by *S. canadensis* and uninvaded plots dominated by three different native grass species: *Phacelurus latifolius*, *Phragmites australis*, and *Imperata cylindrica*.

**Results:**

*S. canadensis* had higher aboveground biomass, higher leaf N, P, and K concentrations, and consequently, a larger macronutrient pool size in the standing biomass. *S. canadensis* also produced more litter with higher N, P, and K concentrations and faster decomposition rates. The *S. canadensis* invasion did not change the total N, P, and K concentration in the topsoil (0–10 cm), but the invasion did increase their availability. The *S. canadensis* invasion did not increase the total soil organic matter (TSOM) content but did increase the soil microbial biomass and the activities of urease, alkaline phosphatase, invertase, amylase, and glucosidase in the topsoil.

**Conclusion:**

The invasion of *S. canadensis* accelerates the macronutrient cycling rate via increases in aboveground productivity and nutrient accumulation in standing biomass, faster nutrient release from litter and higher soil microbial activity. An enhanced nutrient cycling rate may further enhance its invasiveness through a positive feedback on soil processes.

## Introduction

The invasion of exotic plant species has altered ecosystems and caused tremendous ecological and economical loss (Pimentel et al., 2001; Flory and D’Antonio, 2015). Exotic plant invasions can change only plant community productivity (Ehrenfeld, 2003, 2010), but also soil properties such as soil nutrient availability and soil microbial communities (Perkins and Nowak, 2013; Souza-Alonso et al., 2014), Changes in soil properties, in turn, may cause further invasions through positive plant-soil feedbacks (Perkins and Nowak, 2013; Suding et al., 2013; Yelenik and D’Antonio, 2013). Therefore, understanding how exotic plant invasions alter soil properties and their subsequent impacts on further invasion is essential not only for elucidating mechanisms underlying plant invasions but also for their practical management (Daehler et al., 2016).

Soil nutrient availability is one of the most important soil properties frequently altered by plant invasions (Ehrenfeld, 2003). While many studies have shown that invasive plant species increase soil C and N stocks and N, P, and K availability (Ehrenfeld, 2003; Liao et al., 2008; Castro-Díez et al., 2014; Sardans et al., 2017), the patterns and extent of such impacts may vary greatly (Ehrenfeld, 2010). Also, little or lack of effects of plant invasions on soil nutrient availability is reported in some case studies (Vanderhoeven et al., 2006; Stefanowicz et al., 2017; Osunkoya et al., 2017). To explain the variation, it is essential to elucidate mechanisms underlying effects of plant invasions on changes in soil nutrient availability.

The effects of an exotic invasive plant on soil nutrient availability are closely related to nutrient cycling processes, including plant biomass accumulation, litter production and decomposition and soil nutrient transformation (Ehrenfeld, 2010). Increased soil nutrient availability by plant invasions has been frequently associated with high aboveground biomass production (Vanderhoeven et al., 2006) and a high value of some functional traits of invasive plants such as tissue N and P concentrations (Sardans et al., 2017) and litter decomposition rate (Duda et al., 2003; Ehrenfeld, 2003; Ashton et al., 2005; Arthur et al., 2012). However, studies have shown that plant invasions had no or even a negative effect on nutrient concentrations in biomass, litter and soils (Vanderhoeven et al., 2006; Martin et al., 2009) and that litter decomposition rate did not differ between native and non-native plants (Bottollier-Curtet et al., 2011; Jo et al., 2016). Moreover, the effects of plant invasions on soil nutrient availability have been shown to be closely associated with initial nutrient levels of soil (Dassonville et al., 2008; Sardans et al., 2017). Thus, impacts of plant invasions on soil nutrient availability vary with species, functional trait and soil nutrient levels (Dassonville et al., 2008; Slesak et al., 2016). To understand these variations, detailed assessments of nutrient budgets should be conducted to elucidate how exotic species change nutrient cycling between plants and soil (Dassonville et al., 2008).

Several alien species from genus *Solidago*, from North American are found very successful worldwide (Weber, 1998; Szymura and Szymura, 2016). In central Europe, three invaders, *S. canadensis*, *S. gigantea*, and *S. altissima* have been found (Szymura and Szymura, 2013) and it is observed that *S. canadensis* (Canada goldenrod) have became dominant in many sites and caused loss of diversity of native species in Europe (Gusev, 2018). It is also invasive in Asia and Australia (Dong et al., 2006). *S. canadensis* was introduced to China in 1930s as an ornamental flower, and it is the only invasive species from the genus, has spread to the south part of China (Guo et al., 2016) and decreased biodiversity through biotic homogenization (Chen et al., 2013). Its invasion success is associated with the strong plant-soil feedback, which is also found in invasive *S. gigantea* (Scharfy et al., 2010; Schittko and Wurst, 2014; Dong et al., 2017). However, the underlying mechanisms of the plant-soil interactions still need to be elucidated. To date, much attention has been paid to the invasion effects of this species on underground processes, such as soil nutrient transformation (Zhang C.B. et al., 2009; Li et al., 2012) and soil microbial community modification (Li et al., 2012; Wang et al., 2018). However, aboveground processes of *S. canadensis*, especially that of litter decomposition, have not been intensively studied. Litter decomposition was considered as the one of the most important components of invasive species – soil feedback (Gaertner et al., 2014). In the congener species, invasive S. gigantea accelerated nutrient cycling by having much higher aboveground productivity and nutrient pools, and consequent high production of litters (Vanderhoeven et al., 2006), which suggest the roles of aboveground productivity and litter decomposition as a driving force in invasive plant-soil feedback processes.

Up to now, integrated studies on the effects of the *S. canadensis* invasion on aboveground biomass and nutrient accumulation, litter production and decomposition and soil nutrient availability and microbial activity have not been reported. This integrated study is certainly essential to fully understand the mechanisms behind the effects of the *S. canadensis* invasion on soil processes. Although the distribution of *S. canadensis* is not limited to coastal areas, it is observed that *S. canadensis* is steadily invading into coastal grasslands in Eastern China and frequently forms monocultures, with patches size of ca. 10–500 m^2^. We also observed that it is gradually replacing *Imperata cylindrica*, a native grass, in a coastal area. It remains unclear why *S. canadensis* can become so invasive in this particular habitat type. It is reported that *Solidago* species prefers old fields in both its native range as well as in the invaded range (Werner et al., 1980; Szymura and Szymura, 2013), possibly due to less competition from the companion species. Sardans et al. (2017) show that plant invasion is associated with higher plant–soil nutrient concentrations in nutrient-poor environments. Soils in coastal areas are generally deficient in macronutrients and have relatively high salt concentrations (Zhang et al., 2015) and it is possible that the success of *S. canadensis* in the coastal area is associated with the low soil nutrient status and the vegetation characteristics of dominance of grass species tolerant of low nutrient levels in these habitats. In this study, we conducted a field study in a grassland of a coastal area, comparing the difference in the aboveground biomass and nutrient pool size, litter production and decomposition, soil nutrient availability and microbial activity between *S. canadensis* monocultures with adjacent native species stands, dominated by three grasses. We aimed to link aboveground biomass production and belowground soil processes and ask two questions: (1) Does the *S. canadensis* invasion increase the availability of the soil macronutrients, N, P, and K, compared with adjacent native species stands? (2) If it does increase soil nutrient availability, are these effects accompanied by high biomass and nutrient accumulation in species aboveground part, faster litter decomposition and higher soil microbial activities, compared with native species stands? We expected that *S. canadensis* significantly increases the soil nutrient availability, and at the same time, increases aboveground biomass, nutrient pool size, litter amount and decomposition rate and soil microbial availability.

## Materials and Methods

### Study Site

The studied area (center: 121°09′58″ E, 30°19′29″ N, mean height above sea level: 1 m) was originally part of tidal plains and was separated from Hangzhou Bay in 1997, when a dike was built. Therefore, the direct influence of tidal seawater was removed. Thereafter, the land was used as cultivated fields until 2009, when a national wetland park was set up. In the national park, the area has been strictly protected from human disturbance, and the vegetation began as secondary succession into grasslands. The soil water concentration and salt concentration gradually decreased after disconnection with the tidal plain. Presently, the soil is coastal saline and silty clay in texture. The mean soil water content is 14.9–21.5%, and the salt concentration is 0.1–0.5%. The soil pH is greater than 8, and the average available soil N, P, and K contents are 47.3, 5.7, and 92.1 mg/kg, respectively. The area lies in the subtropical monsoon zone; hence, the climate is mild, warm and humid. The duration of summer (June to September) and winter (December to March) is longer than spring (April to May) and autumn (October to November). The annual mean air temperature is 16.0°C and is lowest (3.8°C) in January. The mean rainfall is 1344.7 mm, with peaks in June and September, the annual average sunlight duration is 2038.4 h, and the frost-free period is 244 days.

In the study area, we found the three pioneer communities with different dominant species (Table 1): (i) *Imperata cylindrica* (Poaceae) Community, (ii) *Phacelurus latifolius* Community, (iii) *Phragmites australis* (Poaceae) Community and *Solidago canadensis* (Asteraceae) Community. The vegetation is strictly protected from human disturbance.

**TABLE 1:** Characteristics of the four stand types.

**Stand type**	**Dominant plant species**	**Dominant species height (m)**	**Coverage (%)**	**Shoot density (/m^2^)**	**Major accompanying species**
I	*Solidago canadensis*	1.53	85.1	62.8	*Artemisia lavandulaefolia, Imperata cylindrica*
II	*Phacelurus latifolius*	1.48	81.9	51.4	*Artemisia lavandulaefolia, Glycine soja, Erigeron philadelphicus, Sonchus brachyotus, Imperata cylindrica*
III	*Imperata cylindrica*	0.93	85.1	148	*Melilotus officinalis, Artemisia lavandulaefolia, Sonchus brachyotus, Erigeron annus, Metaplexis japonica, Solidago canadensis*
IV	*Phragmites australis*	1.42	66.8	37.9	*Carex scabrifolia, Aster subulatus, Polypogon fugax, Sonchus brachyotus, Solidago canadensis*

### Aboveground Biomass and Soil Sampling

For each type of plant community with different species, *S. canadensis*, *P. latifolius*, *P. australis, and I. cylindrica*, five plots (3 × 3 m) were set up. To minimize the impacts of possible differences in plot location on explaining the soil and plants nutrient status, the sampling plots were carefully selected. These plots were selected, as the stands growth conditions are very common in the region and can act as representatives for the performance of the species in the studied area. The distances between these sampling plots were limited to 500 m, and they have very similar habitats characteristics (such as elevation and the distance to roads). To take account of the variability in the plant growth and heterogeneity of the soil properties, for each plot, three quadrates (1 × 1 m) determined by square frames were randomly selected within each plot for plant and soil sampling. The data for each plot for analysis is the averaged means of the three quadrates.

In August 2016, for each quadrate, the aboveground parts of the plants, including stems and leaves, were harvested. The soils and the plant materials were sampled once. After harvesting, plant parts from each species were separated. The plant materials were oven dried at 60°C for 72 h, and then, the dry mass was determined. Because dry mass of accompanying species only accounted for a small portion of the total mass of each quadrate (<5%), only dry mass of the dominant species was used in the analysis. At the same time, soils from each quadrate were sampled from the 0–10 cm and 10–20 soil layers with a soil borer (0.04 m in diameter). Four cores were collected at the four corners of respectively and one core at the center of the quadrate and then homogenized them into a single bulk sample for each square, following the method described in Vanderhoeven et al. (2005). These soil samples were transported to the laboratory. The plant samples and soil samples of the three quadrates from the same plot were mixed as one replicate in the latter statistical analysis. Small plant tissues and stones were removed from the samples. Subsamples of the soils were then air-dried until reaching a constant weight and sieved (180 μm) before the nutrient concentration analysis, and the others were kept at 4°C for the analyses of the soil organic carbon content and soil enzyme activity.

### Plant Litter Mass and Decomposition Rate

In each plot, three additional quadrates (1 × 1 m) were randomly set up for litter harvesting. From December to February of 2016, litters of stems and leaves in each quadrate were separately harvested for each species according to the timing of litter production. Then, we pooled together litters of the dominant species within each plot. All litters were dried in air.

Litter decomposition rate of the species was determined *in situ* with litterbag incubations. Recently produced litter was collected and combined within a species type to produce a composite sample. Five replicate samples of air-dried litter (15 g weight and 3 cm length) of each of the four species were placed in litter bags (10 cm × 20 cm, 1 mm mesh) and used to assess the decomposition rate. The in situ decomposition rate measurement was conducted from the end of March to the end of October. After incubation for 6 months, the remaining litter was processed by carefully removing dirt or other contaminants before being dried for the litter mass measurement. The relative litter decomposition rate (%) was determined as the lost litter mass/the initial litter mass × 100%.

### Nutrient Concentrations in the Aboveground Biomass and in Litter

Plant nutrient (N, P, and K) concentrations were determined with the methods described below. The plants materials, including the harvested aboveground leaves and stems, and leaf litter, were ground into powder and passed through a 180 μm sieve. Then, the plant material was digested for 1 h at 200°C and 2 h at 340°C in a mixture of concentrated sulfuric acid and 30% hydrogen peroxide. For plant total N and total P determinations, the Kjeldahl method in a FOSS KJELTEC 2300 Auto Kjeldahl Analysis Equipment (Foss Tecator AB, Hoganas, Sweden) and the colorimetric Mo-blue method (Olsen and Sommers, 1982) were used, respectively, for plant total K, the solution was analyzed in an Inductively Coupled Plasma Optical Emission Spectrometer (Thermo iCAP 7400, Thermo Fisher Scientific, Cambridge, United Kingdom). For each element of N, P, and K, the aboveground nutrient pool was calculated as biomass × nutrient concentration in the biomass.

### Soil Basic Properties, Nutrient Concentrations, and Availability

Soil pH was determined in a soil solution of 1:2.5 (soil:distilled water) with a digital pH meter (PHSJ-3F, NESA Scientific Instrument Co., Ltd., Shanghai, China). The soil water content was calculated as content (%) = (Fresh weight-Dry weight)/(Fresh weight) × 100 by drying three replicate sub-samples of each soil sample at 70°C for 48 h. The soil bulk density and porosity determinations followed the method of Zhu and Zhu (2015).

Total soil N was quantified by use of the Kjeldahl method. Soil mineral N was extracted with 2 mol/L^–1^ KCl, and concentrations of NH_4_^+^-N and NO_3_-N were measured using the phenolate method and cadmium column reduction, respectively. Soil available N, referred to as alkali-hydrolysable N in this study, was assayed using the alkaline-hydrolysis and diffusion method. The available N was reduced to NH_3_ at 40°C for 24 h after adding FeSO_4_ powder and a NaOH solution, and then, the NH_3_ was absorbed using H_3_BO_3_ and titrated using H_2_SO_4_ to determine the NH_4_-N concentration (Wei et al., 2016).

For the total soil P concentration analysis, the soil samples were digested with a mixed solution of H_2_SO_4_-HCLO_4._ Soil available phosphorus was extracted according to Jakmunee and Junsomboon (2009). Total P and available P in the solutions were then determined by the colorimetric Mo-blue method.

For the soil K analysis, soil samples were melted with NaOH following the modified sodium hydroxide fusion method for total soil K analysis (Smith and Bain, 1982). Soil available K was extracted with 1 mol L^–1^ ammonium acetate. Soil total K and available K were both determined by the same instrument described above.

### Soil Organic Matter Fractions and Enzyme Activity

The total soil organic matter (TSOM) concentration was determined by a K_2_CrO_7_-H_2_SO_4_ oxidation procedure (Lu, 1999); soil microbial biomass carbon (MBC) was measured with the fumigation–extraction method (Vance et al., 1987). Soil water-soluble organic carbon (WSOC) was determined following the procedures of Jones and Willett (2006). Soil readily oxidizable carbon (ROC) followed the method in Stianna et al. (2010)

The soil enzymes assayed were catalase, urease, alkaline phosphatase, invertase, amylase, and β-1,4-glucosidase. All the methods followed those described by Guan (1986). The substrates for the analyses of the above enzymes were hydrogen peroxide, urea, disodium phenyl phosphate, sucrose, starch, and carboxyl methylcellulose, respectively.

### Data Analysis

For each nutrient (N, P, and K), the aboveground nutrient pool was calculated as the product of biomass and the concentration in the biomass. For aboveground biomass, N, P, and K concentrations and the pool size in aboveground biomass, plant litter quantity, N, P, and K concentrations in litter and the litter decomposition rate were analyzed in one-way ANOVAs (Analysis of Variances). The N, P, and K concentrations, their availability in soil, and soil enzyme activity were analyzed using two-way ANOVAs, with “species” and “soil layer depth” as the main effects. Since we wanted to emphasize the general impact of species identity on soil properties, the significance of the differences in soil properties from different soil layer depths is not shown. For each plot, the data were averaged from the three quadrates within the plot before analysis. The data were-log transformed when the analysis of the residuals deviated from assumptions of normality and homogeneity. A *post hoc* mean separation was performed by Duncan’s multiple range test at *P* < 0.05.

## Results

### Soil Properties and Nutrient Concentrations

The invasion of *S. canadensis* had significant effects on the soil pH and water content (*P* < 0.05, Table 2). The soil pH and water content were lowest in the soils from *S. canadensis* in both soil layers (0–10 cm and 10–20 cm). There were no significant differences in the soil bulk density and porosity between the three native species (*P* > 0.05, Table 2).

**TABLE 2:** Basic properties at the two soil depths in the community dominated by each of the four plant species.

**Soil property**	**Depth (cm)**	***S. canadensis***	***P. latifolius***	***I. cylindrica***	***P. australis***
pH	0-10	8.17±0.03⁢a	8.47±0.04⁢c	8.28±0.01⁢b	8.57±0.04⁢d
	10-20	8.36±0.05⁢a	8.57±0.08⁢b	8.38±0.02⁢a	8.69±0.02⁢b
Water content (%)	0-10	19.05±1.8⁢a	29.62±1.5⁢b	25.19±2.1⁢b	29.47±2.35⁢b
	10-20	17.47±1.9⁢a	27.54±1.4⁢b	26.81±1.7⁢b	25.63±2.32⁢b
Bulk density (g/cm^–3^)	0-10	0.91±0.09⁢a	0.90±0.05⁢a	0.85±0.03⁢a	0.99±0.06⁢a
	10-20	1.02±0.05⁢a	0.88±0.09⁢a	0.90±0.08⁢a	1.02±0.06⁢a
Porosity (%)	0-10	65.67±3.26⁢a	66.17±1.89⁢a	68.02±1.25⁢a	62.56±1.13⁢a
	10-20	61.52±1.91⁢a	66.96±3.29⁢a	66.29±3.13⁢a	61.74±2.50⁢a

**Data are means ± SE. Within the same row, means sharing the same letters are not different at P = 0.05 (by Tukey test).**

The invasion of *S. canadensis* did not significantly affect the soil total N, P, and K concentrations (*P* > 0.05), but it did affect the availability of N, P, and K to plants in the soils (Table 3). Specifically, the invasion of *S. canadensis* increased soil NH_4_-N and NO_3_-N concentrations for the both soil layers (*P* < 0.05). The soil available P and available K concentrations in the 0–10 cm soil layer from *S. canadensis* were higher than that from *I. cylindrica*, but lower than *P. australis*, and was not different from that of *P. latifolius* (Table 3); in the 10–20 cm soil layer, the soil available N, P, and K concentrations from *S. canadensis* were all intermediate in the four species (Table 3).

**TABLE 3:** N, P, and K concentrations at the two soil depths in the community dominated by each of the four plant species.

**Soil nutrient**	**Depth (cm)**	***S. canadensis***	***P. latifolius***	***I. cylindrica***	***P. australis***
Total N (g/Kg)	0-10	0.50±0.03⁢a	0.47±0.07⁢a	0.38±0.03⁢a	0.42±0.02⁢a
	10-20	0.35±0.04⁢a	0.38±0.02⁢a	0.27±0.01⁢a	0.39±0.07⁢a
Total P (g/Kg)	0-10	0.63±0.02⁢a	0.58±0.01⁢a	0.60±0.01⁢a	0.57±0.03⁢a
	10-20	0.62±0.03⁢a	0.57±0.01⁢a	0.59±0.01⁢a	0.58±0.01⁢a
Total K (g/Kg)	0-10	5.56±0.82⁢a	5.77±0.29⁢a	4.66±0.24⁢a	5.67±0.18⁢a
	10-20	5.22±0.66⁢a	5.94±0.27⁢a	4.56±0.27⁢a	5.49±0.16⁢a
NH_4_^+^-N (mg/Kg)	0-10	7.89±1.12⁢b	4.56±0.36⁢a	4.69±0.29⁢a	4.30±0.27⁢a
	10-20	6.59±0.64⁢b	4.24±0.47⁢a	4.35±0.47⁢a	3.47±0.11⁢a
NO_3_^–^-N (mg/Kg)	0-10	4.63±0.45⁢b	2.61±0.12⁢a	2.54±0.08⁢a	2.36±0.07⁢a
	10-20	3.32±0.19⁢b	2.77±0.05⁢a	2.82±0.11⁢a	2.83±0.09⁢a
Available N (mg/Kg)	0-10	74.7±10.0⁢b	43.4±8.7⁢a	31.8±5.2⁢a	37.4±4.6⁢a
	10-20	27.0±5.7⁢a	41.7±7.06⁢b	21.1±1.4⁢a	24.3±3.0⁢a
Available P (mg/Kg)	0-10	7.68±1.59⁢bc	5.05±0.94⁢ab	3.06±0.26⁢a	9.95±0.60⁢c
	10-20	5.54±0.99⁢b	5.34±1.19⁢b	2.81±0.22⁢a	10.2±0.5⁢c
Available K (mg/Kg)	0-10	110.5±16.2⁢b	86.3±14.7⁢ab	49.3±4.8⁢a	125.5±17.7⁢b
	10-20	73.6±17.5⁢ab	95.4±14.4⁢bc	49.7±4.3⁢a	147.8±13.5⁢c

**Data are means ± SE. Within the same row, means sharing the same letters are not different at P = 0.05 (by Tukey test).**

### Soil Organic Matter Content and Enzyme Activity

**FIGURE 1:**
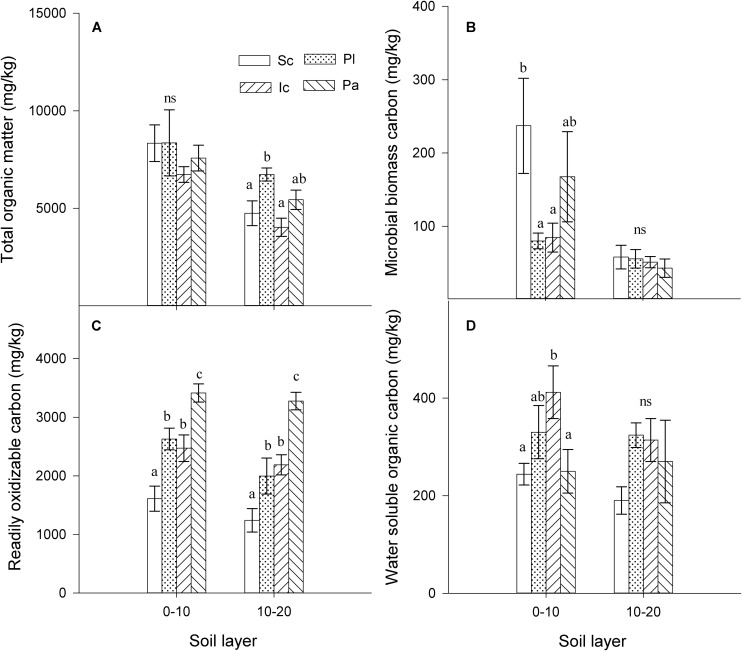
Soil organic matter content in the soils from different species. **(A)** total soil organic matter (TSOM), **(B)** microbial biomass carbon (MBC), **(C)** readily oxidizable carbon (ROC), and **(D)** water soluble organic carbon (WSOC). Sc, *S. canadensis*; Pl, *P. latifolius*; Ic, *I. cylindrical*; Pa, *P. australis*). Soils are sample from two layers (0–10 cm and 10–20 cm). Bars are means ± standard error. Different letters indicate significant differences between the species for the parameters within the same soil layer (*P* < 0.05). ns, non-significant, *P* > 0.05.

The invasion of *S. canadensis* did not affect the total TSOM concentration in the 0–10 cm soil layer (Figure 1A), but it increased the MBC concentration (*P* < 0.05, Figure 1B) and decreased the ROC concentration (*P* < 0.05, Figure 1C) and WSOC concentration (*P* < 0.05, Figure 1D) in the 0–10 cm soil layer. The TSOM concentration in the 10–20 cm soil layer under *S. canadensis* was significantly lower than that of *P. latifolius*, but similar to that of *I. cylindrica* (Figure 1A). The soil MBC concentration was not significantly different between the four plant species in the 10–20 cm soil layer (*P* < 0.05, Figure 1B). The invasion of *S. canadensis* also decreased the soil ROC concentration and WSOC concentration in the 10–20 cm soil layer, although this decrease was not significant for the WSOC concentration (Figure 1D, *P* > 0.05).

The invasion of *S. canadensis* increased the activities of urease, alkaline phosphatase, invertase, amylase and glucosidase in the 0–10 cm soil layer (*P* < 0.05), but not in the 10–20 cm soil layer (*P* > 0.05). Soil catalase activity under *S. canadensis* was similar to that of *P. latifolius* and higher than that of *I. cylindrica*, and there were no significant differences between the three species in the 10–20 cm soil layer (Table 4).

**TABLE 4:** Enzyme activity at the two soil depths in the community dominated by each of the four plant species.

**Soil enzyme**	**Depth (cm)**	***S. canadensis***	***P. latifolius***	***I. cylindrica***	***P. australis***
Catalase (ml/g^–1^)	0-10	9.83±0.08⁢a	9.83±0.17⁢a	9.26±0.21⁢a	9.39±0.45⁢a
	10-20	8.89±0.70⁢a	9.94±0.03⁢a	9.41±0.53⁢a	9.34±0.50⁢a
Urease (μg/g^–1^)	0-10	316.6±32.0⁢c	165.3±28.6⁢b	145.9±33.8⁢ab	72.8±11.3⁢a
	10-20	156.5±41.8⁢a	120.9±39.8⁢a	109.8±37.9⁢a	47.7±2.9⁢a
Alkaline phosphatase (μg/g^–1^)	0-10	353.7±70.8⁢b	203.1±53.4⁢a	191.4±34.8⁢a	103.8+15.6a
	10-20	117.2±45.2⁢a	104.4±30.1⁢a	73.9±22.7⁢a	48.7±12.4⁢a
Invertase (mg/g^–1^)	0-10	6.96±1.69⁢b	2.62±0.76⁢a	3.14±0.76⁢a	1.12±0.21⁢a
	10-20	2.85±1.28⁢a	1.12±0.42⁢a	0.61±0.06⁢a	0.65±0.10⁢a
Amylase (μg/g^–1^)	0-10	269.6±18.8⁢a	187.0±17.9⁢a	169.1±34.7⁢a	174.2±33.9⁢a
	10-20	172.6±20.9⁢a	160.7±23.6⁢a	134.2±19.7⁢a	154.9±35.1⁢a
Glucosidase (μg/g^–1^)	0-10	281.9±68.1⁢b	102.0±29.1⁢a	67.2±11.1⁢a	49.5±10.0⁢a
	10-20	76.9±35.4⁢a	46.2±11.3⁢a	21.3±1.6⁢a	28.5±5.3⁢a

**Data are means ± SE. Within the same row, means sharing the same letters are not different at P = 0.05 (by Tukey test).**

### Aboveground Biomass, Nutrient Concentrations, and Nutrient Pool

**FIGURE 2:**
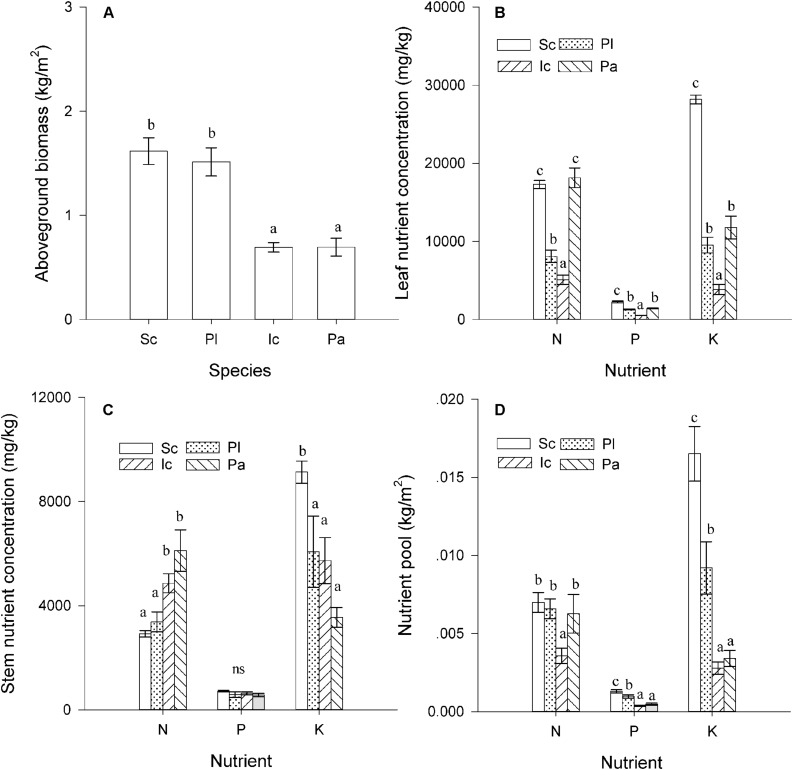
Nutrients accumulation in the aboveground stands. **(A)** Aboveground biomass (Kg/m^2^). **(B)** Nutrient (N, P, and K) concentrations (mg/g) in leaves. **(C)** Nutrient (N, P, and K) concentrations (mg/g) in stems **(C)**. **(D)** Nutrient (N, P, and K) pools (g/m^2^) in aboveground biomass (g) of the four dominant species. Sc, Pl, Ic, and Pa stand for *S. canadensis*, *P. latifolius*, *I. cylindrical*, and *P. australis*, respectively. Bars and vertical lines are means ± SE. Different letters indicate significant differences between the species (*P* = 0.05).

The four species differed significantly in their aboveground biomass, nutrient concentrations, and nutrient pool sizes (Figure 2). The total amount of aboveground biomass of *S. canadensis* was similar to that of *P. latifolius*, but much higher than that of *P. australis* and *I. cylindrica* (Figure 2A, *P* < 0.05). *S. canadensis* also had the highest P and K concentrations in leaves (Figure 2B, *P* < 0.05); the N concentration in the N concentration in *S. canadensis* leaves was significantly higher than that of *P. latifolius* and *I. cylindrical* (Figure 2B, *P* < 0.05), but similar to that of Figure 2B (*P* > 0.05). In stems, only the K concentration was much higher in *S. canadensis* stems (Figure 2C, *P* < 0.05). The N concentration in *S. canadensis* stems was similar to that of *P. latifolius* and lower than that of *I. cylindrica* and *P. australis* (Figure 2C, *P* < 0.05). The P concentrations in stems of the three native species were not significantly different from each other (Figure 2C, *P* > 0.05). The N, P, and K pool sizes in the aboveground mass were all highest for *S. canadensis* and lowest for *I. cylindrica* (Figure 2C, *P* < 0.05).

### Litter Mass, Nutrient Concentrations, and Decomposition Rate

**FIGURE 3:**
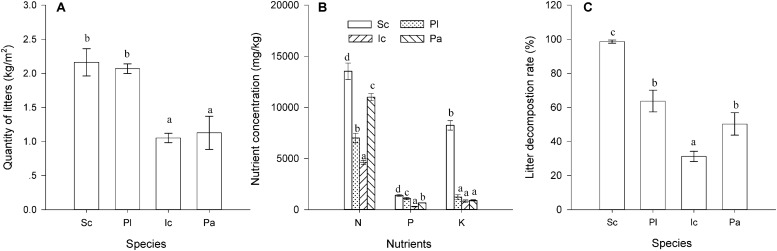
Litter quantity, nutrient concentration and decomposition rate of the species. **(A)** Leaf and stem litter mass (g); **(B)** Nutrient (N, P, and K) concentrations (mg/kg), and **(C)** Decomposition rate (the ratio of decomposed litter to the original litter mass, %). Sc, Pl, Ic, and Pa stand for *S. canadensis*, *P. latifolius*, *I. cylindrical*, and *P. australis*, respectively. Bars and vertical lines are means ± SE. Different letters indicate significant differences between the species (*P* = 0.05).

The four species also differed significantly in their litter quantity, litter nutrient concentrations and litter decomposition rate (*P* < 0.05, Figure 3). The total leaf and stem litter mass of *S. canadensis* was similar to that of *P. latifolius*, but much higher than that of *I. cylindrica* and *P. australis* (Figure 3A, *P* < 0.05). The N, P, and K concentrations in litter were highest in *S. canadensis* and lowest in *I. cylindrica* (Figure 3B, *P* < 0.05). The litter decomposition rate was also much higher in *S. canadensis* than in the other three species (Figure 3C, *P* < 0.05)

## Discussion

Our study indicated an accelerated nutrient cycling rate by the invasion of *S. canadensis* into the grassland, which remarkably differentiated itself from the three native grass species. *S. canadensis* possessed the features of higher nutrient availability and higher soil microbial activity in the soils, higher plant productivity and a larger nutrient pool size in the plants, a faster nutrient release rate by litter decomposition, suggesting that these features may be closely associated with each other.

### Increased Soil Nutrient Availability and Soil Microbial Activity by *S. canadensis* Invasion

Consistent with many studies which show that invasive species tends to increase soil nutrient status (Ehrenfeld, 2003; Vanderhoeven et al., 2005; Yelenik and D’Antonio, 2013) the *S. canadensis* invasion increased the availability of soil N, P, and K. However, S. canadensis invasion did not increase the total nutrient content, suggesting it probably only affected nutrient transformations within soils and the available nutrient exchange between the plants and the soil. The results are not completely consistent with those reported by Zhang C.B. et al. (2009) who found that the *S. canadensis* invasion decreased total nitrogen, total phosphorus, NO_3_-N, available phosphorus contents but increased the NH_4_-N content. This finding suggests that effects on soil nutrient availability may vary substantially. Various factors may control the direction and extent of modification of soil nutrient availability by *S. canadensis* invasion.

Firstly, the impact on soil nutrient availability may be linked to the differences in functional traits related to nutrient cycling between the invasive species and the uninvaded vegetation being compared (Castro-Díez et al., 2014). Forbs (in this case *S. canadensis*), diverge from grasses in leaf nutrient concentrations and litter decomposition rate. In our study, the native dominant species in the coastal area are all grasses, which are generally characterized by low leaf nutrient concentrations and slow leaf litter decomposition rates, possibly adaptations to the low soil nutrient levels, while the higher nutrient concentration and faster litter decomposition rate of the invasive *S. canadensis* may be phylogenetically determined. One of the factors determining decomposition rate is litter nutrient concentration. Higher nutrients nutrient concentrations in both living leaf tissues and litter suggest that *S. canadensis* is probably both highly nutrient demanding and luxurious in nutrient utilization, as it still leaves high concentrations of nutrients in the litter and consequently accelerates the nutrient cycling rate, which is also found in another study (Zhang et al., 2016). The decomposition rate of leaf litter is also affected by other leaf attributes, such as the concentration of recalcitrant materials (like lignin) and structural functional traits (like specific leaf area) (Austin and Ballaré, 2010; Jo et al., 2016). The leaves of *S. canadensis* are indeed thinner and softer than those of the other three species, which may also contribute to its fast decomposition rate.

Secondly, the other factor explaining the increase in soil nutrient availability is the increased soil microbial activity, because soil microbial activity determines the nutrient transformation rate. The *S. canadensis* invasion increased the soil MBC concentration and the activity of some key enzyme, which indicated that both the abundance of activity of soil microorganisms increased. Other studies also show that the *S. canadensis* invasion alters the soil microbial community structure (Li et al., 2012) and increases the nutrient transformation rate (Zhang C.B. et al., 2009; Li et al., 2012). The most important factors in explaining the enhancement of soil microbial activity are increased litter input and a fast litter decomposition rate, which supply more substrates to feed a large microbial population. Studies also suggest that *S. canadensis* may alter the soil microbial community structure by releasing some species-specific compounds into soils via root exudates. Several studies report that *S. canadensis* is allelopathic in the invaded soil (Abhilasha et al., 2008; Zhang S.S. et al., 2009). Root exudates may affect nutrient transformations directly or by soil biota. These results suggest that there are strong species-specific interactions between plant species and the microbial community in the soil and species-specific allelopathic compounds may alter the soil microbial community and nutrient availability.

Thirdly, the impact of invasive species on the soil nutrient status is significantly influenced by the initial soil conditions, which tends to homogenize soil nutrient level (Vanderhoeven et al., 2005; Dassonville et al., 2008; Osunkoya et al., 2017). The increased N, P, and K availability in *S. canadensis*-invaded soils in this study may indicate the relatively low nutrient availability in the early stage of plant community succession in this newly reclaimed coastal area (less than 20 years). The soil available N and K concentrations were in the range of 31.7–43.3 mg/kg and 49.2–125.5 g/kg, respectively, in the topsoil, and both fell into the lower range of soil nutrient concentrations in the study of Dassonville et al. (2008). However, the concentration of available P in topsoil was 5.1–9.9 mg/kg, which was much higher than 0.5–2.5 mg/kg reported by Dassonville et al. (2008), suggesting that the potential of increase in P availability is much higher in the soils in our study.

### Increased Aboveground Biomass and Nutrient Accumulation by *S. canadensis* Invasion

The first important factor for accelerated nutrient cycling was increased biomass accumulation by *S. canadensis*. Invasive plant species usually grow faster (Grotkopp and Rejmánek, 2007), have a larger plant size, and accumulate more plant biomass than the species from the community in which they invade (van Kleunen et al., 2010). The greater aboveground biomass of *S. canadensis* was attributable to the larger shoot height and higher density of the plant, and these characteristics enable the *S. canadensis* plants to be more competitive. The populations in our study may have evolved to be more competitive than their native populations, although a study found that plants from invasive *S. canadensis* populations in Europe tended to grow smaller than their counterparts from the native populations in America (van Kleunen and Schmid, 2003)

What makes *S. canadensis* more productive than the two grasses? Higher nutrient concentration may explain higher productivity. The higher P and K concentrations in the tissues may contribute to the high productivity of *S. canadensis*. We observed that the K concentration was remarkably higher, ca. 139–633% in leaves and 50–157% in stems, in *S. canadensis* than in the other three species. K is one the most demanded nutrients for plant growth and plays fundamental roles in plant functions (Armengaud et al., 2009; Hu et al., 2018), especially in alleviating the detrimental effects of abiotic stresses and improving water use efficiency (Cakmak, 2005). A growing number of studies have shown that K may contribute to the success of exotic plant invasions, depending on the initial soil K availability (Sardans and Peñuelas, 2015). Higher K concentrations were also observed in invasive species compared with adjacent native species stands. A high K accumulation capability was also found in invasive *S. gigantean* (Vanderhoeven et al., 2006), a congeneric species of *S. canadensis*, *Alternathera philoxeroides* (Song and Su, 2013), and *Eichhornia crassipes* (Zhou et al., 2007). In *A. philoxeroides*, the accumulation of K enhances drought resistance (Song and Su, 2013). These studies suggest that the accumulation of K may enhance the competitive ability of an invasive species under stressful conditions. P is one of the most limiting nutrients for plant growth (Vance et al., 2003). Higher leaf P concentrations may also contribute to the higher growth capacity of *S. canadensis*. Leaf N concentrations were also higher in *S. canadensis* than in *P. latifolius* and *I. cylindrica*, suggesting that N accumulation may also account for the higher growth.

The observed higher P and K concentrations in both leaves and stems and the higher N concentration in leaves may be due to the inherent high nutrient uptake capacity by the species, but may also be due to the high P and K availability in the soils under *S. canadensis*. It is therefore critical to differentiate the nutrient uptake capacity of the invasive and native species under homogenous nutrient conditions. A recent study showed that *S. canadensis* was capable of utilizing insoluble phosphorus (Wan et al., 2018), suggesting that root exudates, such as organic acids from *S. canadensis*, might be involved in releasing P and K from insoluble sources (Bais et al., 2006).

Our study only addresses the effects of *S. canadensis* invasion on nutrient cycling in the coastal grassland in one region. As *S. canadensis* invades habitats with diverse vegetations, soil types and climate regimes, and previous study show that impacts of alien invasive plants on soil nutrients may highly depend on initial site conditions (Dassonville et al., 2008; Osunkoya et al., 2017), therefore the information from our study on the invasion effects is limited. Secondly, in our study only aboveground biomass and nutrient accumulation are considered, while the information on roots and rhizomes not considered. Thirdly, the soil properties and nutrient status were only analyzed from only one sampling in the growing season, while these properties may vary with and this may limit full understanding the invasion effects of the species. Lastly, elucidation of general patterns of the invasion effects of *S. canadensis* demand integration of both field studies and controlled pot studies.

### Implications for Further Invasion of *S. canadensis*

Positive invasive plant-soil feedbacks have been assumed an important mechanism for exotic plant invasions (Suding et al., 2013). Further invasion of *S. canadensis* may be promoted by increased soil nutrient availability. Pot studies did find significant plant-soil feedback effects by *S. canadensis* invasion, which leads to subsequent changes of its own growth and its competitive ability against natives, but these effects vary with the identity of the native species (Schittko and Wurst, 2014; Dong et al., 2015, 2017). The variation of the competition effects is possibly because species from different functional groups may have different responses to soil nutrient availability. Since it has been stated that the *S. canadensis* invasion also significantly changes the soil microorganism community and functions, there might be strong nutrient-microorganism interactions in the plant-soil feedback from *S. canadensis*. The role of soil nutrients in plant-soil feedbacks needs to be studied further.

## Conclusion

In summary, the *S. canadensis* invasion increases nutrient accumulation in the plants and nutrient availability in the soil. These invasion effects are very likely due to its greater biomass accumulation together with greater litter production, faster litter decomposition and consequent higher soil microbial activity. The increase of soil nutrient concentrations and microbial activity probably promote the further invasion of *S. canadensis* through a positive feedback loop and is likely one important mechanism of its success in the invaded region. The driving forces of accelerating nutrient cycling by *S. canadesis* need to be further examined in controlled studies.

## Author Contributions

X-QY and MW conceived the study, designed the work, and conducted the data analysis. X-QY and Y-NY carried out field work and laboratory analysis. X-QY wrote the manuscript, with contributions from all the other authors. MW revised the final draft. F-hY helped in preparing the manuscript and in interpretation of the analyses during constructive discussions.

## Conflict of Interest Statement

The authors declare that the research was conducted in the absence of any commercial or financial relationships that could be construed as a potential conflict of interest.

## Abbreviations

MBC: microbial biomass carbon
ROC: readily oxidizable carbon
TSOM: total soil organic matter
WSOC: water-soluble organic carbon.
